# Influenza A virus selectively elevates prostaglandin E_2_ formation in pro-resolving macrophages

**DOI:** 10.1016/j.isci.2023.108775

**Published:** 2023-12-26

**Authors:** Paul M. Jordan, Kerstin Günther, Vivien Nischang, Yuping Ning, Stefanie Deinhardt-Emmer, Christina Ehrhardt, Oliver Werz

**Affiliations:** 1 Department of Pharmaceutical/Medicinal Chemistry, Institute of Pharmacy, Friedrich Schiller University Jena, Philosophenweg 14, 07743 Jena, Germany; 2 Jena Center for Soft Matter (JCSM); Friedrich Schiller University Jena, Philosophenweg 7, 07743 Jena, Germany; 3 Institute of Medical Microbiology, Jena University Hospital, Am Klinikum 1, 07747 Jena, Germany; 4 Section of Experimental Virology, Institute of Medical Microbiology, Center for Molecular Biomedicine (CMB), Jena University Hospital, Hans-Knoell-Str. 2, 07745 Jena, Germany

**Keywords:** Immunology, Virology

## Abstract

Respiratory influenza A virus (IAV) infections are major health concerns worldwide, where bacterial superinfections substantially increase morbidity and mortality. The underlying mechanisms of how IAV impairs host defense remain elusive. Macrophages are pivotal for the innate immune response and crucially regulate the entire inflammatory process, occurring as inflammatory M1- or pro-resolving M2-like phenotypes. Lipid mediators (LM), produced from polyunsaturated fatty acids by macrophages, are potent immune regulators and impact all stages of inflammation. Using LM metabololipidomics, we show that human pro-resolving M2-macrophages respond to IAV infections with specific and robust production of prostaglandin (PG)E_2_ along with upregulation of cyclooxygenase-2 (COX-2), which persists after co-infection with *Staphylococcus aureus*. In contrast, cytokine/interferon production in macrophages was essentially unaffected by IAV infection, and the functionality of M1-macrophages was not influenced. Conclusively, IAV infection of M2-macrophages selectively elevates PGE_2_ formation, suggesting inhibition of the COX-2/PGE_2_ axis as strategy to limit IAV exacerbation.

## Highlights

• IAV selectively induces PGE_2_ formation in human M2-but not in M1-macrophages
• Besides PGE_2_, no other lipid mediator was increased in M2-macrophages by IAV
• IAV fails to induce cytokine/interferon release and functionality of macrophages
• Elevation of PGE_2_ in M2 by IAV persists after co-infection with *S. aureus*

## Graphical abstract

**Figure:**
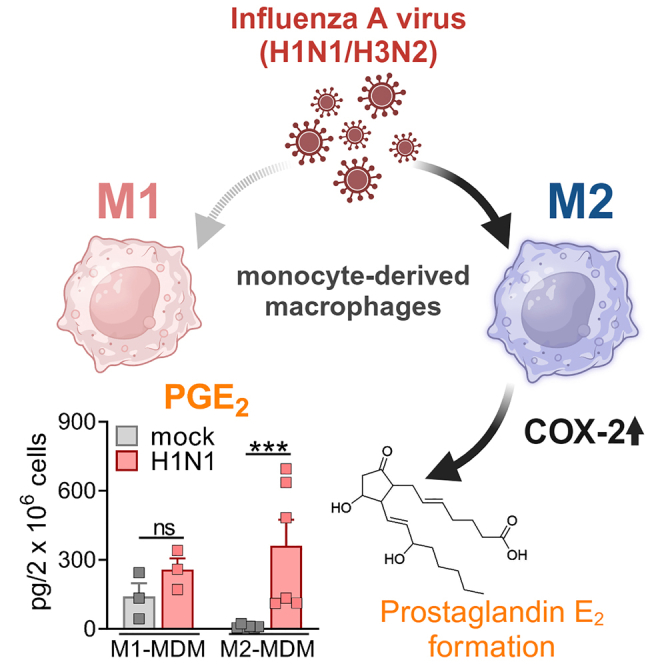

## Introduction

Infections with influenza A virus (IAV) are a leading cause of morbidity and mortality of pneumonia in both children and adults, accompanied by suppressed and dysregulated host immune functions by multiple mechanisms.^1^ However, autopsy case reports revealed that >90% of deaths during the 1918 influenza pandemic proceeded from secondary pneumonia due to bacterial superinfections.^2^^,^^3^ *Staphylococcus aureus* (*S. aureus*) is one of the most prominent colonizing pathogens in this respect, which can either be persistent or non-persistent,^4^ ranging from skin and soft tissue infections to life-threatening disease states, such as bacteremia, septicemia, or pneumonia.^5^^,^^6^ The simultaneous occurrence of various pathogens can exuberate pathological effects in affected organs. Thus, superinfection of IAV-infected patients with *S. aureus* leads to increased inflammatory lung damage.^7^ The underlying mechanism of how IAV substantially impairs host defense against bacteria (e.g., *S. aureus*) and thus facilitates bacterial infections is still elusive.^8^

The acute inflammatory response is a major protective mechanism of the host to eliminate invading pathogens. Pathogens are detected mainly by pattern-recognition receptors (PRRs) of tissue-resident innate immune cells, followed by specific release of pro-inflammatory cytokines (e.g., TNF-α, IL-1β, and IL-6) and type I-III interferons (IFN).^9^ The PRRs for IAV infections recognize viral RNA, while the main pathogen-associated marker pattern (PAMP), are Toll-like receptors (TLRs), retinoic acid-inducible gene-I (RIG-I), and the nucleotide-binding oligomerization domain (NOD)-like receptor family pyrin domain containing 3 (NLRP3) domain.^10^^,^^11^ Besides cytokines, also the production of polyunsaturated fatty acid (PUFA)-derived lipid mediators (LMs) that play crucial roles in host defense by tightly regulating inflammatory and immune responses, are formed in mammals during acute infections with various pathogens.^12^ Prominent LMs in the initiation phase of inflammation are eicosanoids derived from arachidonic acid (AA) such as prostaglandins (PG) and leukotrienes (LT), formed by the cyclooxygenase (COX) and 5-lipoxygenase (LOX) pathways, respectively.^13^ In the resolution phase of infectious diseases, specialized pro-resolving mediators (SPM) are produced by various LOXs as key enzymes, which are LM that terminate acute inflammation and promote the resolution of inflammation and the return to tissue homeostatic states.^14^^,^^15^

It was shown that mice infected with highly pathogenic 1918 pandemic H1N1 and H5N1 IAV strains exhibited increased numbers of macrophages and neutrophils in the lungs compared to mice infected with low-pathogenic viruses.^16^ Detection of replicating viruses in lung macrophages led to the conclusion that macrophages are one of the key target cells for IAV.^16^^,^^17^^,^^18^ Macrophages are central in regulating the resolution of acute inflammation by mechanisms such as bacterial clearance and promoting tissue regeneration and the return to homeostasis.^19^ Differential polarized M1- and M2-like phenotypes of monocyte-derived macrophages (MDM) play prominent roles in the defense against pathogens and are well-coordinated with each other. Notably, inflammation-initiating mediators such as cytokines, chemokines, LTs, and PGs are primarily formed by M1-, whereas M2-like macrophages produce SPMs and anti-inflammatory cytokines.^20^^,^^21^ We recently found that bacteria-induced production of LOX-derived LTs and SPMs depends on bacterial pathogenicity,^21^ and that distinct bacterial exotoxins activate differential LOX pathways in innate immune cells.^22^^,^^23^ Along these lines, cytokines and eicosanoid storms are associated with severe IAV infections and are risk factors for superinfection with pathogens such as *S. aureus*.^24^^,^^25^ Here, we evaluated the response of human M1- and M2-MDMs upon exposure to *S. aureus* or H1N1 alone, but also aimed at mimicking the frequently occurring IAV*/S. aureus* superinfections by co-infecting H1N1-predisposed macrophages with *S. aureus*.^26^^,^^27^ We found that the responses of M1- and M2-like phenotypes differ upon IAV infection, with most striking impact of H1N1 on PGE_2_ formation in M2-MDM along with elevating COX-2 induction, besides rather moderate changes on other LM and inflammatory signaling proteins like cytokines and IFN.

## Results

### Impact of H1N1 or *S. aureus* on secretion of cytokines, chemokines, and interferons from human M1- and M2-MDMs

**Figure 1:**
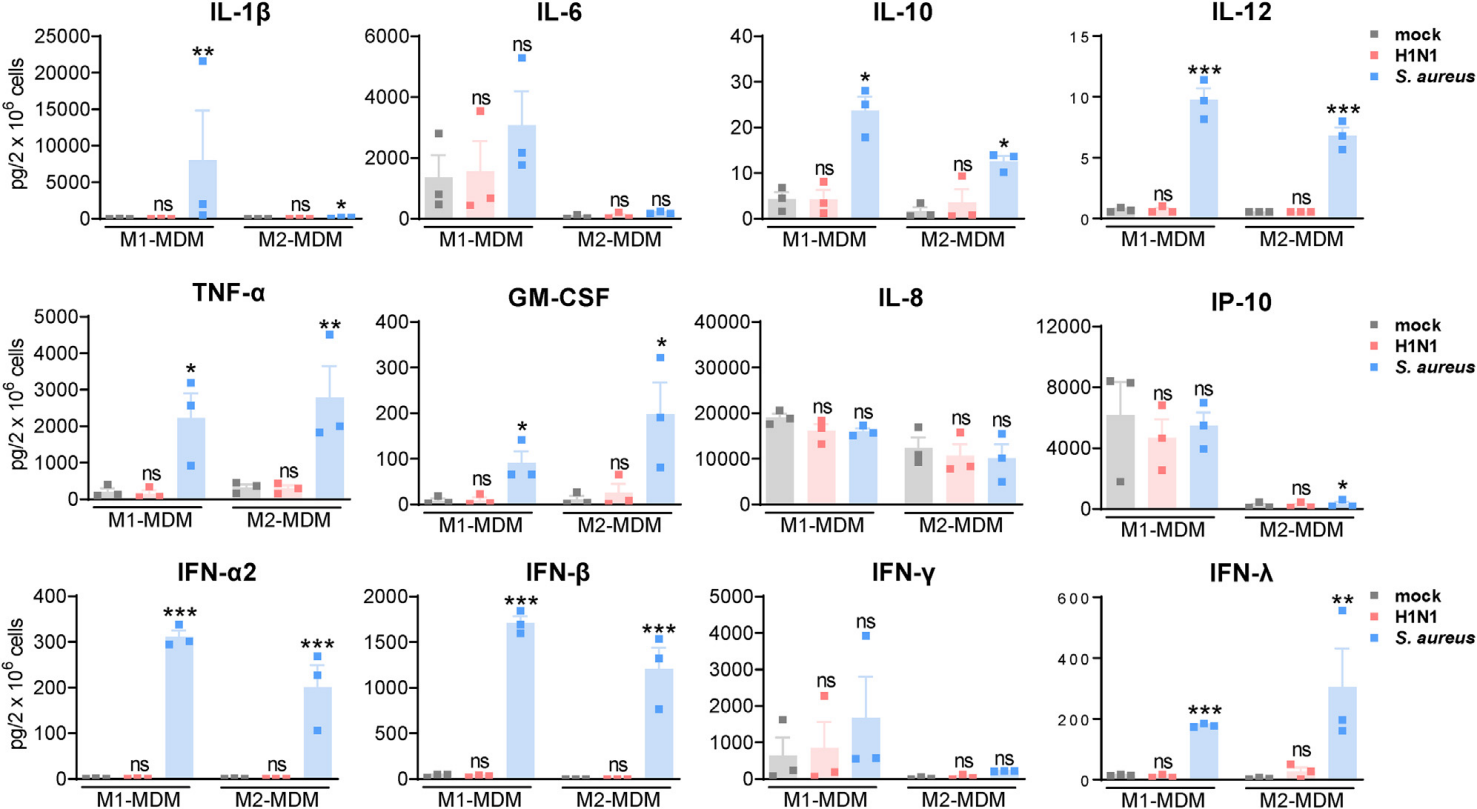
*S. aureus*- and H1N1-induced secretion of cytokines in human M1-and M2-MDMs Human M1- or M2-MDM (2 × 10^6^ cells) were infected with IAV H1N1 (PR8; MOI = 5) or mock for 30 min, then washed, and further incubated for 4 h at 37°C. In parallel, mock-treated cells after washing were exposed to *S. aureus* (MOI = 10) and also incubated for 4 h at 37°C. Supernatants were then collected and secreted cytokines were assessed via flow cytometry. Data are shown as means + SEM and single values, n = 3, given as pg/2 × 10^6^ cells, for mock-, H1N1- or *S. aureus*-treated M1- and M2-MDMs. Data were log-transformed for statistical analysis; ∗p < 0.05; ∗∗p < 0.01; ∗∗∗p < 0.001; versus mock-infected MDMs; one-way ANOVA with Dunnett’s multiple comparisons test.

In order to study innate host cell responses to IAV H1N1 or *S. aureus* infections, especially inflammation-related protein and LM signatures, primary M1- and M2-MDMs were infected according to general standard infection models with IAV.^16^^,^^17^^,^^28^ For this purpose, the well characterized and suitable A/Puerto Rico/8/34 H1N1 strain, isolated from humans and propagated on Madin Darby canine kidney (MDCK) cells, was employed that induces reasonable immune responses in murine and human cell cultures. We assessed M1-/M2-MDM responses due to H1N1 or *S. aureus* infection after short-term incubation periods (<5 h), in order to exclude detrimental effects on the MDM due to excessive viral or bacterial replication. Thus, H1N1 were added to MDMs at a multiplicity of infection (MOI) of 5, and after 30 min of internalization, MDMs were washed and incubated for another 4 h. In parallel, *S. aureus* at an MOI of 10 was added to MDMs for 4 h to evoke host cell responses; this rather low MOI is less cytotoxic than an MOI of 50 that was used in previous studies as stimulus for induction of LM formation within 1.5–3 h.^22^ Production of cytokines, chemokines, and IFNs as inflammation-related protein mediators was measured in the supernatant by using the human antivirus response panel LEGENDplex. H1N1 did not significantly induce cytokine, chemokine, or IFN release from M1- or M2-MDMs (Figure 1). In contrast, upon exposure to *S. aureus,* M1-MDMs strongly released the pro-inflammatory cytokines IL-1β, IL-12, TNF-α, and granulocyte-macrophage colony-stimulating factor (GM-CSF) (but not IL-6) as well as the anti-inflammatory IL-10, while the chemokines IL-8 and IP-10 were not or hardly elevated (Figure 1). In M2-MDM, *S. aureus* caused overall less pronounced effects, markedly inducing only the release of IL-10, IL-12, TNF-α, and GM-CSF, with minor impact on IL-1β and IL-6 as well as on the chemokines IL-8 and IP-10. Note that the latter chemokines were secreted in high amounts already from resting M1-MDM. Also the absolute amounts of some cytokines and of IFN-γ that inhibits IAV attachment and replication,^29^ also differed between the MDM phenotypes. Moreover, after *S. aureus* (but not H1N1) exposure of both M1- and M2-MDM, a strong IFN response was observed, with substantial production of type I IFN-α2 and IFN-β, and type III interferon IFN-λ but no significant elevation of IFN-γ. Possibly, longer incubation of IAV-exposed MDM is required to evoke type I IFN-α2 and -β release, as observed for epithelial cells where IFN-β was secreted 8 to 12 h upon IAV exposure.^30^ Together, exposure of M1/M2-MDM to H1N1 failed to elevate cytokine, chemokine, and IFN production, while *S. aureus* strongly increased all analyzed cytokines and type I/III IFNs in M1-MDM, and, except IL-1β and IL-6, also in M2-MDM.

### H1N1 specifically evokes prostaglandin E_2_ production in human M2-MDMs and induces COX-2 expression

**Figure 2:**
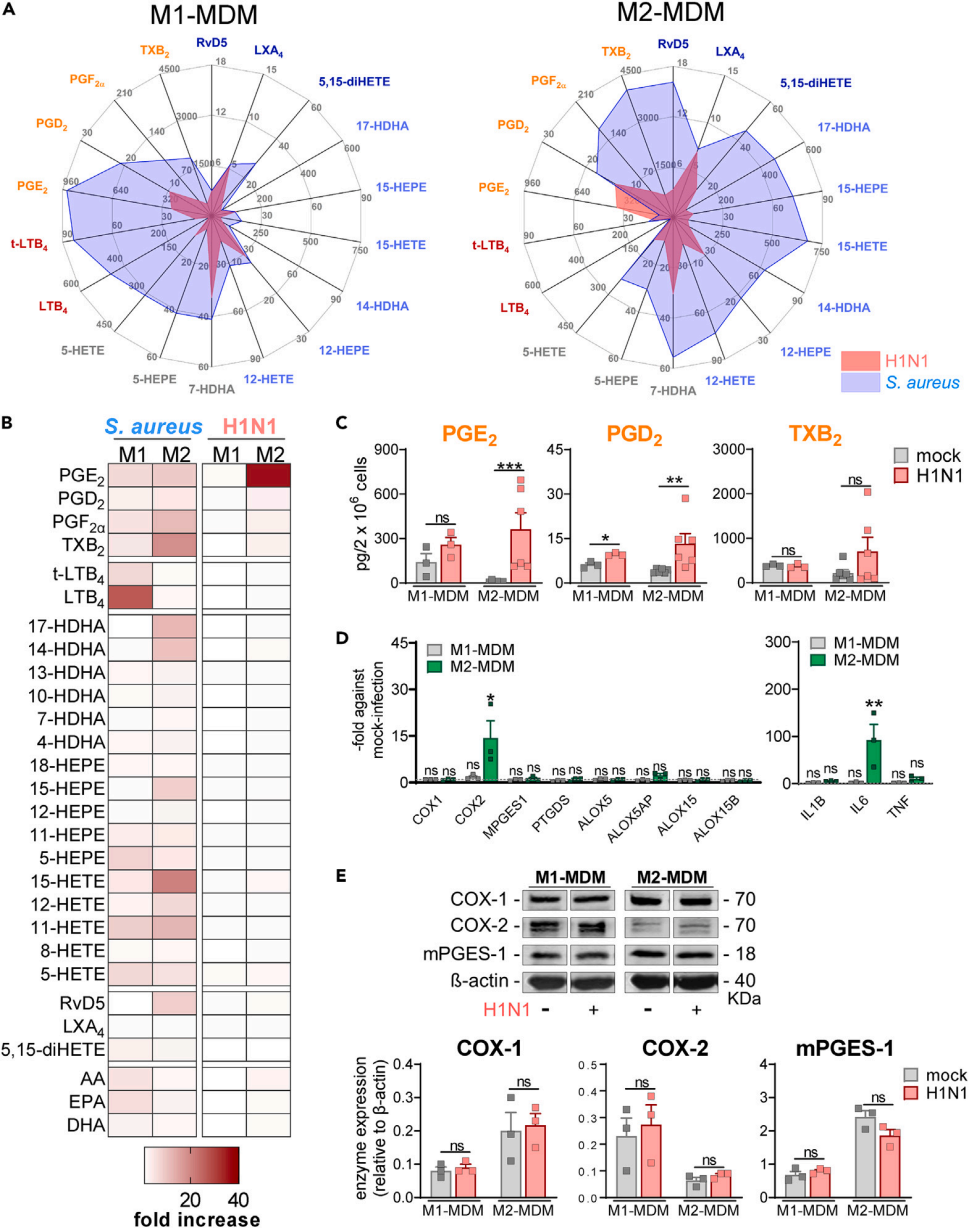
H1N1 specifically evokes PGE_2_ production in human M2-MDMs (A–C) Human M1- or M2-MDMs (2 × 10^6^ cells) were infected with IAV H1N1 (PR8; MOI = 5) or mock for 30 min, then washed, and further incubated for 4 h at 37°C. In parallel, mock-treated cells after washing were exposed to *S. aureus* (MOI = 10) and also incubated for 4 h at 37°C. Formed LMs in the supernatants were analyzed by UPLC-MS-MS. (B and C) Data, given as pg/2 × 10^6^ cells, n = 3 for M1-MDMs, n = 6 for M2-MDMs, are shown for H1N1- or *S. aureus*-treated MDMs in radar plots (A), or presented in heatmaps showing the -fold change to mock control (B). In (C), COX-mediated LMs, given as pg/2 × 10^6^ MDMs, are shown as bar charts; data are means + SEM with single values; n = 3 for M1-MDMs, n = 6 for M2-MDMs. Data were log-transformed for statistical analysis; ∗p < 0.05; ∗∗p < 0.01; ∗∗∗p < 0.001; versus mock-infected MDMs; ratio-paired t-test. (D and E) M1-and M2-MDMs were incubated infected with H1N1 (PR8; MOI = 5) or mock for 30 min, then washed, and further incubated for 4 h at 37°C. (D) mRNA levels of indicated genes were analyzed by real-time PCR; data, means + SEM, are shown in bar charts as -fold of control with single values, ∗p < 0.05; ∗∗p < 0.01; versus mock-infected MDMs; ratio-paired t-test. n = 3. (E) Cells were immunoblotted for COX-1, COX-2, and mPGES-1 protein expression by western blot and normalized to β-actin. Exemplary results and densitometric analysis are shown. Data are presented as means + SEM for M1- and M2-MDMs; ratio-paired t-test. n = 3. See also Figures S1 and S2, Table S1, and Data S1.

By employing ultra-performance liquid chromatography-tandem mass spectrometry (UPLC-MS-MS)-based targeted LM metabololipidomics, we investigated the differential LM signature profiles (25 LMs and three PUFAs) generated and released by M1- and M2-MDMs after exposure to H1N1 or *S. aureus*. As previously reported, *S. aureus* evokes differential LM profiles in human M1- and M2-MDMs, where M1 produced mainly COX-derived PGs and 5-LOX-mediated LTs, while M2-MDMs formed 15-LOX-derived SPM and their precursors,^22^ confirmed in the present study (Figure 2A). For comprehensive LM analysis, we utilized meaningful LM radar plots (Figure 2A) and heat maps (Figure 2B), revealing that compared to *S. aureus,* H1N1 induces LM signatures (i) to an overall much lower extent in both macrophage phenotypes and (ii) with a different pattern of individual LM (Figures 2A and 2B). Thus, in contrast to *S. aureus,* H1N1 neither elevated formation of 5-LOX-mediated LTs nor of 12/15-LOX-derived SPMs (resolvin (Rv)D5 or lipoxin (LX)A_4_) or SPM precursors (17-hydroxydocosahexaenoic acid (HDHA), 15-hydroxyeicosapentaenoic acid (HEPE) or 15-hydroxyeicosatetraenoic acid (HETE)), indicating that H1N1 did not activate LOX signaling pathways (Figures 2A and 2B). In M1-MDM, H1N1 did not markedly increase any of the 25 LM and the three PUFAs versus mock infection (Figure 2B). However, In M2-MDMs, H1N1 specifically elevated COX-derived PGE_2_ formation (approx. 40-fold), and also the production of other COX-derived PGs, i.e., PGD_2_ and PGF_2α_, was significantly increased (approx. 6- to 8-fold) in M2-MDMs, and to a minor extent in M1-MDMs, while TXB_2_ was the overall most abundant COX product in M1- and M2-MDM (Figure 2C) in line with previous MDM studies^12^^,^^20^^,^^21^ but not enhanced by H1N1 in either phenotype (Figure 2C). By analysis of LM signatures of human macrophages infected with the IAV H3N2 subtype (A/Wisconsin/67/2005), we found similar effects as observed with H1N1 (Figure S1). We next investigated mRNA expression levels of LM-biosynthetic enzymes and pro-inflammatory cytokines (Figure 2D). We found that H1N1 significantly increased mRNA levels of COX-2 in M2-MDMs (about 15-fold). Of interest, expression of IL-6 mRNA but not of IL-1β and TNFα was significantly increased in M2-MDMs, whereas M1-MDMs did not respond in this respect (Figure 2D). In contrast to the increased COX-2 mRNA levels, the absolute amounts of COX-1, COX-2, and mPEGS-1 proteins were not altered within 4 h post H1N1 infection of M1- or M2-MDMs (Figure 2E and Data S1). Furthermore, we found that COX activity and COX-1/2 protein levels were not further increased within 6 h post H1N1 infection compared to 4 h, indicating that formation of COX products, especially of PGE_2_, takes place shortly after H1N1 exposure, as initial response of the M2-MDMs (Figure S2 and Data S1). Absolute data for mock-, single H1N1- or *S. aureus*-infected M1- and M2-MDMs, given in pg/2 × 10^6^ cells, are shown in Figures 4B and S3.

### Co-infection of MDMs with H1N1 and *S. aureus* has a minor impact on bacterial or viral titers

**Figure 3:**
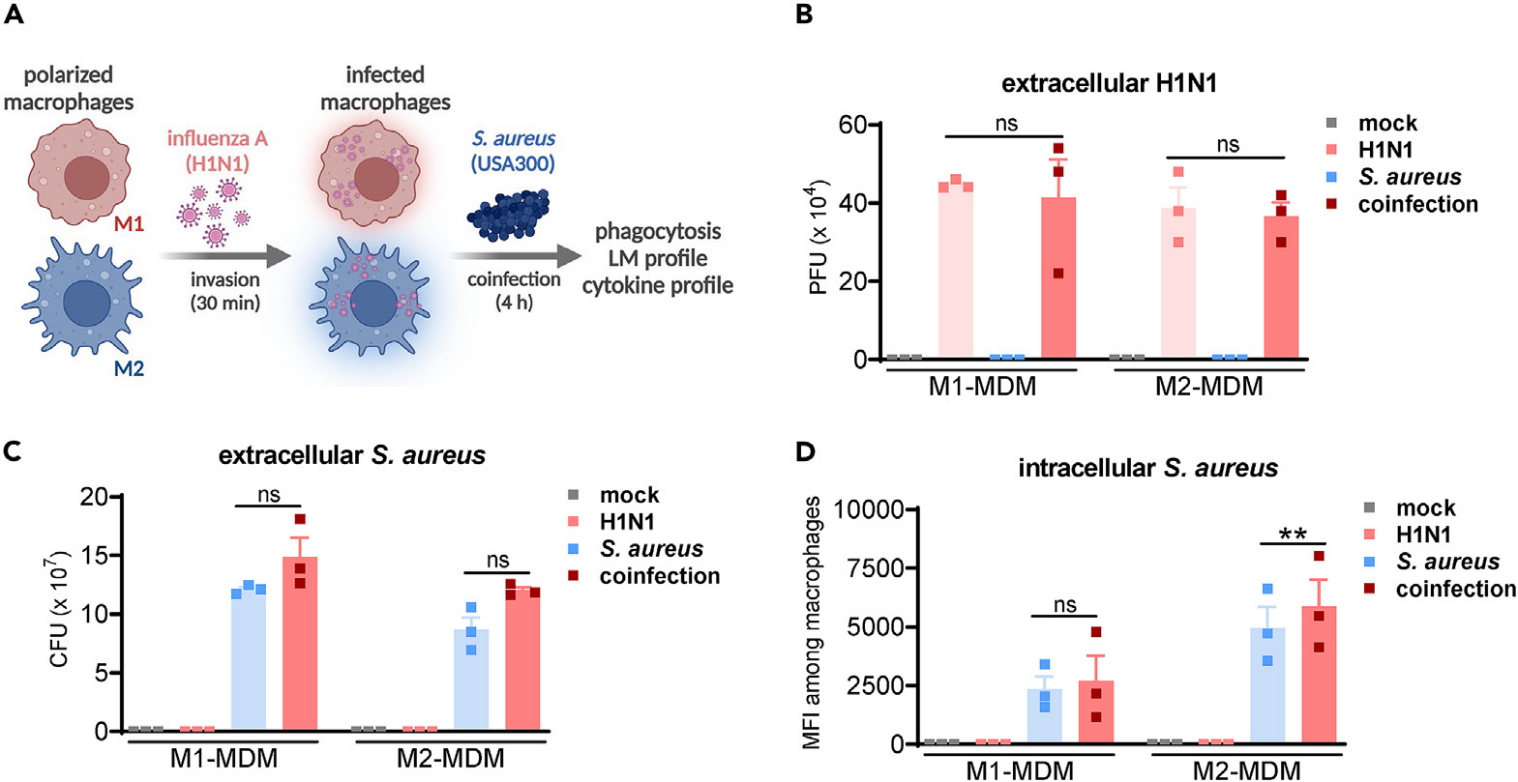
Impact of co-infection of MDMs with H1N1 and *S. aureus* on bacterial or viral titers (A) Scheme depicting the experimental settings of the *in vitro* co-infection model. (B and C) Human M1- or M2-MDMs (2 × 10^6^ cells) were infected with H1N1 (PR8; MOI = 5) or mock for 30 min, then washed, and coinfected with *S. aureus* (MOI = 10) or mock for 4 h at 37°C. Supernatants were collected to determine extracellular viral and extracellular bacterial titers. (B) Extracellular H1N1 are represented by plaque-forming units (PFU), data are shown in bar charts as mean + SEM with single values, ns = not significant, ratio-paired t-test; H1N1-infected MDMs versus co-infected MDMs, n = 3. (C) Extracellular *S. aureus* are represented by colony-forming units (CFU), data are shown in bar charts as mean + SEM with single values, ns = not significant, ratio-paired t-test; *S. aureus*-infected MDMs versus co-infected MDMs, n = 3. (D) Human M1- or M2-MDM (2 × 10^6^ cells) were infected with H1N1 (PR8; MOI = 5) or mock for 30 min, washed, and then co-infected with GFP-expressing *S. aureus* (MOI = 10) or mock for 4 h at 37°C. Cells were detached and GFP-expressing *S. aureus* in the MDMs were measured by flow cytometry. Data are presented as mean fluorescence intensity (MFI) of M1- and M2-MDMs. Results are given as means + SEM with single values in bar charts; n = 3; ∗∗p < 0.01, ratio-paired t-test; *S. aureus* infected MDMs versus co-infected MDMs.

Influenza-associated bacterial co-infections contribute to immune disorders, including failed antibacterial immune response to clear microbial infection,^8^ therefore, we studied the effect of co-infection on immune functions of human MDMs. We investigated whether co-infection of MDMs with both H1N1 and *S. aureus* impacts the load of each pathogen versus single infection. M1- and M2-MDMs were infected with H1N1 (MOI 5) or kept untreated for 30 min, each, cells were washed and then infected with *S. aureus* (MOI 10) or kept untreated for 4 h, each (Figure 3A). We first analyzed the extracellular numbers of plaque-forming units (PFU) and colony-forming units (CFU) to determine viral and bacterial loads in the extracellular environment (supernatants) of MDMs, respectively (Figures 3B and 3C). Neither viral (Figure 3B) nor bacterial titers (Figure 3C) were significantly affected upon co-infection compared to single-infected MDMs. To investigate intracellular bacterial loads, we used gene-modified *S. aureus* expressing GFP to analyze fluorescent contents in the MDMs after infection, and we found that the mean fluorescent intensity in M2-MDMs was slightly increased upon co-infection compared to single-infection, whereas the MFI in M1-MDMs was unaffected, suggesting increased bacterial phagocytosis of H1N1-predisposed M2-MDMs (Figure 3D); whether or not this elevation is due to PGE_2_ remains to be investigated.

### Impact of co-infection of *S. aureus*-treated MDMs with H1N1 on the release of inflammation-related mediators

**Figure 4:**
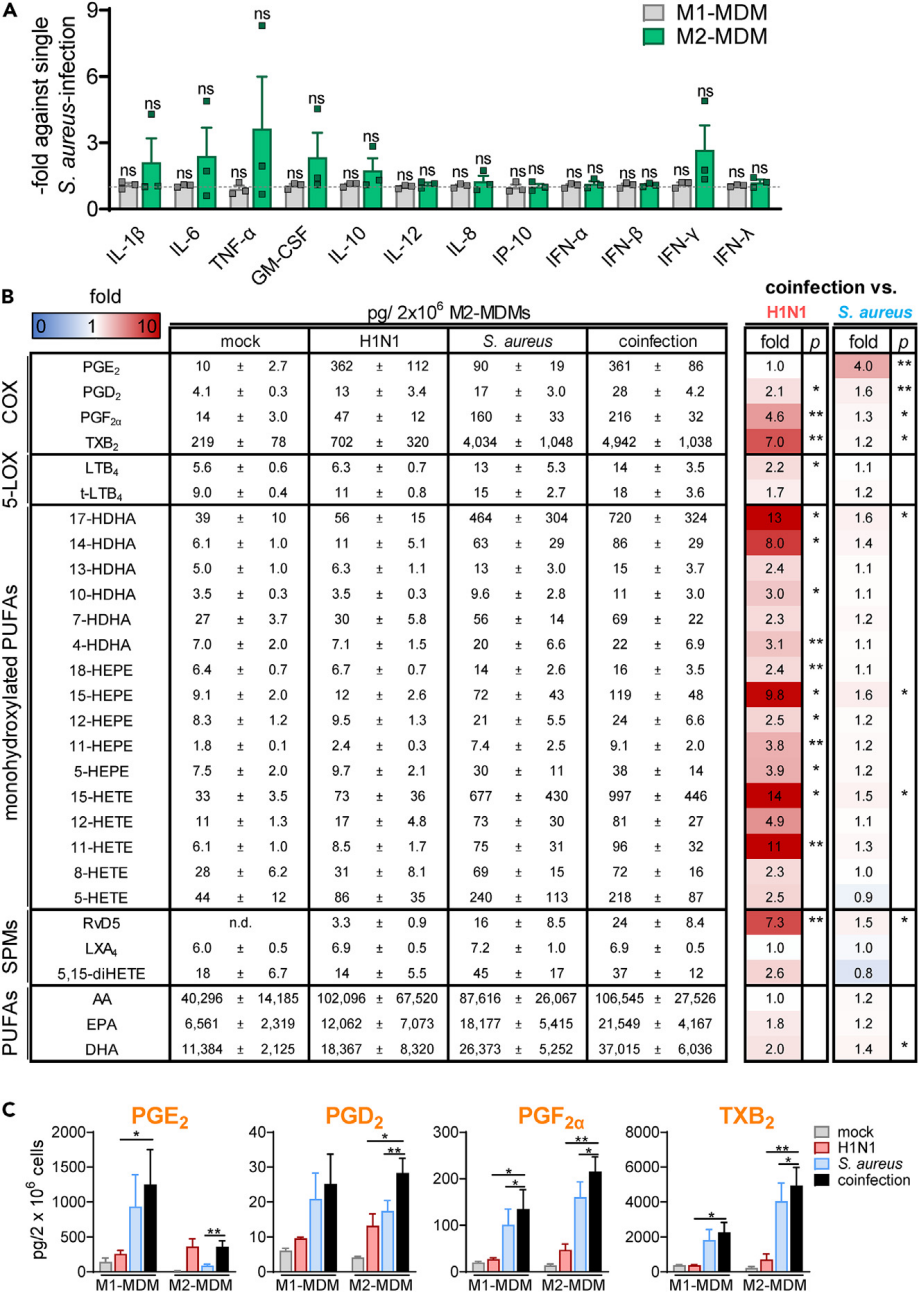
Impact of co-infection of *S. aureus*-treated MDMs with H1N1 on the release of inflammation-related mediators (A and B) Human M1- or M2-MDMs (2 × 10^6^ cells) were infected with H1N1 (PR8; MOI = 5) or mock for 30 min, washed, and then treated with *S. aureus* (MOI = 10) or mock for 4 h at 37°C, as indicated. (A) Supernatants were collected to assess secreted cytokines via flow cytometry. Results of co-infection with H1N1 are shown as -fold change against infection with *S. aureus* alone, given as means + SEM with single values. Raw data were log-transformed for statistical analysis; ns = not significant, ratio-paired t-test; *S. aureus*-infected MDMs versus coinfected MDMs, n = 3. (B and C) Released LMs from M2-MDMs in the supernatants were assessed by UPLC-MS-MS. LM formed by MDM upon infection with mock, single H1N1, single *S. aureus*, and co-infection with H1N1 and *S. aureus* are shown in pg/2 × 10^6^ cells as mean ± SEM, and the fold change of H1N1/*S. aureus*-treated samples versus H1N1 single or *S. aureus* single infection are given and visualized in a heatmap (B), data for mock-, H1N1- and *S. aureus*-treated cells are taken from experiments shown in Figures 2A–2C; COX product formation is visualized in bar charts (C). Data were log-transformed for statistical analysis; ∗p < 0.05; ∗∗p < 0.01; ratio-paired t-test; coinfected MDMs versus H1N1-infected MDMs or *S. aureus*-infected MDMs, n = 3 for M1-MDMs, n = 6 for M2-MDMs. See also Figure S3.

To examine the inflammatory status of H1N1-predisposed MDMs after infection with *S. aureus*, we determined cytokine, chemokine, and IFN levels in the supernatants compared to single *S. aureus* infection. We found that especially in M2-MDMs, pro-inflammatory cytokines such as IL-1β, IL-6, and TNF-α but also the type II interferon IFN-γ are elevated by tendency, without changes in M1-MDMs (Figure 4A). We also analyzed the impact of H1N1 on the LM signatures of MDMs co-infected with *S. aureus*. Note that *S. aureus* causes massive formation of various COX- and LOX-derived LM in MDMs, due to robust LM pathway induction by released exotoxins.^21^^,^^22^ In M1-MDMs, co-infection with H1N1 led to only moderate elevation of *S. aureus*-induced LM release for all determined LM classes (around 10–30% higher versus single-infection with *S. aureus*), reaching significance solely for 11-HETE and 13-HDHA, while the PUFA levels were not altered. However, by comparison of the H1N1-infected cells versus cells that were coinfected with *S. aureus*, increased LM production was observed due to the impact of the bacteria (Figures 4C and S3). In contrast, in M2-MDM, H1N1-predisposal caused strong increases of certain LMs, especially PGE_2_ (up to 402% versus *S. aureus* single-infected MDMs) and other COX-derived PGs. While 5-LOX products were hardly elevated, 15-LOX-mediated LM such as the SPM precursor 17-HDHA, 15-HEPE, and 15-HETE, and the SPM RvD5 were significantly increased in M2-MDMs due to additional H1N1, although to a minor degree (≤164%) as compared to PGE_2_ (Figures 4B and 4C). Furthermore, compared to single H1N1 infection, the co-infection with H1N1 and *S. aureus* led to increased LM formation overall, except for PGE_2_ (and LXA_4_), indicating that H1N1 is the predominant elicitor for PGE_2_ formation in M2-MDMs (Figures 4B and 4C).

## Discussion

Here, we demonstrate that infection of human MDMs with an IAV H1N1 subtype *in vitro* selectively and strongly elevates formation of PGE_2_ in M2-MDM but not in M1-MDM. In contrast, 5-LOX-derived pro-inflammatory LTs or pro-resolving 15-LOX-derived LM and also cytokine/interferon release was not altered in either MDM phenotype. Such selective elevation of PGE_2_ production by IAV is in contrast to the broad COX- and LOX-activating effects caused by human pathogenic bacteria (i.e., *E. coli* or *S. aureus*) in MDM, implying that different mechanisms of LM induction by IAV are operative.^21^^,^^31^ M1-MDMs, with superior COX-2/mPGES-1 expression over M2-MDM, possess higher capacities to produce PGE_2_ upon exposure to bacterial pathogenicity,^21^ but upon infection with H1N1, the PGE_2_ levels of IAV-stimulated M2-MDM surpass those of the M1 counterpart. This effect might be of particular relevance for *in vivo* situations where M2 macrophages dominate over the M1 phenotype. As PGE_2_ was shown to induce the switch from pro-inflammatory toward inflammation-resolving LM,^32^^,^^33^ it is tempting to speculate that also the IAV-mediated induction of PGE_2_ formation in pro-resolving M2 macrophages might contribute to such LM class switching. Our data show that the selective induction of PGE_2_ in M2-MDMs by H1N1 is still evident after co-infection with *S. aureus* that utilizes exotoxins to elicit the formation of broad LM profiles in these cells.^22^ The antigens and molecular mechanisms of how IAV causes such selective PGE_2_ induction, even on top of exotoxin-stimulation, are unknown and require further investigations.

PGE_2_ is a crucial mediator that induces fever in virus-infected organisms and regulates viral replication,^34^^,^^35^ activating several G-protein-coupled receptors (GPCR), namely EP1-4 which mediate pro- and anti-inflammatory effects of PGE_2_. Our finding that PGE_2_ is specifically produced after IAV infection is consistent with observations from IAV-infected mice *in vivo*,^36^ especially in macrophages,^37^ but also with results from other airway-related virus infections, such as respiratory syncytial virus (RSV) in epithelial cells^38^ or SARS-CoV-2 in humans.^39^ It was shown before that IAV rapidly elevates the COX-2 mRNA levels in human lung epithelial cells peaking at 2–4 h but later on decline,^34^ which fits to the elevated mRNA levels of COX-2 in MDM at 4 h in our study. Moreover, a shift toward pro-inflammatory COX-2 expression was also seen in human alveolar epithelial cells and PBMCs of IAV-infected patients.^40^ Lee et al. found differences in the induction of COX-2 in macrophages by different IAV strains, where the avian influenza H5N1 was more robust as compared to H1N1 subtype.^40^ We tested IAV H1N1 and H3N2 and found that these evoke similar responses in MDM, indicating specific mechanisms to induce PGE_2_, which suggests an IAV class effect.

It was shown before that secretory factors in the supernatants of IAV-infected macrophages induce mRNA expression of COX-2 and of other pro-inflammatory cytokines such as TNF-α, IL-6, and IL-1β in A549 cells.^40^ We found similar up-regulatory effects of IAV on COX-2 and IL-6 mRNA levels in M2-MDMs, while M1-MDMs were unaffected in this respect. Possible reasons for these differences between the phenotypes could be: (i) activation of TLRs and/or PRRs that are present on M2-but not on M1-MDMs, (ii) pre-activation of PRRs in M1-MDMs due to lipopolysaccharide (LPS) treatment during polarization and, thus, lack of further responsiveness as compared to M2-MDM, (iii) higher susceptibility of M2-MDM for IAV invasion, or (iv) production of M2-MDM-specific mediators which cause COX-2 and IL-6 induction.^41^^,^^42^ Based on our results, at least a higher susceptibility of M2-MDMs for IAV invasion can be excluded as reason, since the H1N1 viral burden of M1-and M2-MDMs was about the same. It is intriguing that the substantial and selective increase of PGE_2_ was not connected to elevated mPGES-1 mRNA or protein levels, neither in M1- nor in M2-MDMs. Among the three PGES isoforms, the mPGES-1 is an inducible isoform that is frequently coupled to the induction of COX-2.^43^ Like for COX-2, M1-MDMs express also high levels of mPGES-1, in contrast to the M2-phenotype,^44^ but in M2-MDMs only COX-2 expression (but not mPGES-1) was elevated, at least on the mRNA level. Interestingly, while COX-2 mRNA was significantly increased in M2-MDMs by H1N1, elevated translation toward COX-2 protein expression was not readily evident. We reported earlier that IAV could destabilize COX-2 mRNA levels in human epithelial cells after 2 to 4 h and thus only transiently elevated COX-2 protein.^34^ It seems that IAV-induced elevation of COX-2 protein in M2-MDM is transient as well, which might explain why the protein levels of COX-2 appeared not altered in MDMs at 4 and 6 h versus uninfected cells. Finally, IAV infections may promote additional events that specifically favor the coupling of COX-2 with PGES isoforms to stimulate PGE_2_ formation on the enzyme activation level; increased AA substrate supply can be excluded as levels of free AA and of other AA-derived LOX products were not elevated. Our data show that infection of MDMs with H1N1 does not increase the secretion of inflammation-related cytokines/IFN (including IL-6) regardless of the phenotype, yet the mRNA of IL-6 was selectively increased in M2-MDMs. This indicates that the translation into IL-6 protein was comparatively limited at least within the short time frame of only 4 h. Such translation can be stimulated by low amounts of LPS or bacterial infection, which potentiates cytokine release.^45^^,^^46^ Along these lines, our data from the co-infection experiments using *S. aureus* and H1N1 showed that in the presence of bacteria, at least some pro-inflammatory cytokines (i.e., IL-1β, IL-6, and TNF-α) and type II interferon IFN-γ are elevated on the protein level by H1N1 in M2-MDMs by tendency. A similar impact of *S. aureus* was observed for LM formation. Thus, compared to the strong increase of PGE_2_ by H1N1 in M2-MDMs, only PGD_2_ but no other LM was elevated in cells infected with IAV alone. However, when co-infected with *S. aureus,* besides elevating PGE_2_ and PGD_2_, IAV caused increased formation of RvD5 and of 15-LOX-derived 17-HDHA, 15-HEPE, and 15-HETE in M2-MDMs, albeit only 1.5- to 1.6-fold as compared to PGE_2_ that was elevated by 4-fold.

Bacterial superinfections are characterized by the loss of epithelial barrier function and altered innate immune functions leading to an overall disturbed host immune response.^47^ Another detrimental effect of IAV is the predisposition of the lung endothelium, causing leakage after exposure to *S. aureus*. PGE_2_ is a likely candidate as respective mediator, resulting in acute respiratory distress syndrome (ARDS), the predominant cause of death in patients with bacterial superinfections.^48^^,^^49^ Since alveolar macrophages in the airways feature an M2-like phenotype, the strong elevation of PGE_2_ that we observed in M2-MDMs after IAV, with or without *S. aureus*, might be of pathophysiological relevance for ARDS.

PGE_2_ can affect cytokine production during the inflammatory process, acting as a highly potent immunomodulatory mediator.^50^ In view of the robust PGE_2_ elevation and the potent pyrogenic features of PGE_2_, the COX-2 is a potential therapeutic target in order to reduce PGE_2_ levels in the infected tissue. For example, treatment of IAV-infected mice with NSAIDs to lower PGE_2_ levels by COX-2 inhibition reduced pathology,^51^^,^^52^ while the administration of PGE_2_ reversed this phenotype.^37^ Moreover, IAV induced less severe illness in COX-2^−/−^ compared to wild-type and to COX-1^−/−^ mice, suggesting that COX-2 deficiency is beneficial, whereas COX-1 deficiency is detrimental to the host during influenza virus infection.^53^ This supports specific inhibition of the COX-2 pathway as pharmacological strategy. However, COX inhibition to lower PGE_2_ levels in IAV-infected patients, as commonly practiced with NSAIDs like ibuprofen or aspirin to reduce fever, is still under debate. For example, PGE_2_ may have anti-viral potential visualized by diminished viral replication rates in A549 cells.^34^

Together, our results demonstrate that the H1N1 IAV evokes robust and selective biosynthesis of PGE_2_ in human M2-MDMs as a rapid response mechanism while cytokine/IFN release and formation of other LM was essentially unaltered. Interestingly, upregulation of PGE_2_ in M2-MDM persists after co-infection with *S. aureus,* although other LM, such as the SPM RvD5 and its precursor 17-HDHA, were increased as well. Notably, M1-MDMs, as primarily anti-microbial macrophage phenotype, did not respond to IAV infection, regardless of co-infection with *S. aureus*, neither in terms of cytokine/IFN nor LM formation.

### Limitations of the study

A limitation of this work is the investigation of isolated macrophages in monocultures infected with pathogens, while the aspect of the epithelial layer is missing but may provide a more comprehensive analysis of the host response to infection. Also, the impact of the robustly generated PGE_2_ by M2-MDMs on the biological outcome of IAV or superinfected organisms *in vivo* remains to be investigated, especially responses of primary human alveolar macrophages or other primary M2-like cells. Furthermore, we investigated the first/immediate response of M1-and M2-MDMs on viral infections within only 4 or 6 h, neglecting replication effects of IAV themselves at later time points due to technical/experimental issues. It also remains challenging to study different primary macrophage populations from tissues with mixed M1- and M2 phenotype. Comparative analysis of M2-MDMs with human alveolar macrophages, the first responders in the lung airway upon IAV infection, may further support the pathophysiological relevance of our findings. But also studies with alveolar macrophages from rodents, for example, may help to confirm the selective PGE_2_ induction by IAV in such cells. Also, more detailed analysis is needed to specify this effect on *Orthomyxoviridae* overall by comparing influenza A, B, and C viruses, and the influence of hemagglutinin and neuraminidase and their antigenic variations due to mutation, specific for the antigenic drift, remain to be investigated. Future studies aiming at evaluating the involvement of TLRs (i.e., TLR-3 and -7, specific for IAV infections), inflammasome activation, or NF-κB, together with translation toward *in vivo* IAV infection models, are planned.

## STAR★Methods

### Key resources table

**Table:** 

REAGENT or RESOURCE	SOURCE	IDENTIFIER
**Antibodies**		

IRDye 680LT goat anti-mouse	Li-Cor Biotechnology	Cat#926-68020; RRID:AB_10706161
IRDye 800CW goat anti-rabbit	Li-Cor Biotechnology	Cat#926-32211; RRID:AB_621843
mouse monoclonal anti-β-actin	Cell Signaling	Cat#3700; RRID:AB_2242334
rabbit monoclonal anti-COX-2	Cell Signaling	Cat#12282; RRID:AB_2571729
rabbit polyclonal anti-COX-1	Cell Signaling	Cat#4841; RRID:AB_2084807
rabbit monoclonal anti-COX-2	Cell Signaling	Cat#12282; RRID:AB_2571729
rabbit monoclonal anti-mPGES-1	Abcam	Cat#ab180589

**Bacterial and virus strains**		

*S. aureus*/USA300	kindly provided by Dr. Lorena Tuchscherr, University Hospital Jena, Germany	N/A
*S. aureus*/USA300/GFP	kindly provided by Dr. Oliwia Makarewicz, University Hospital Jena, Germany	N/A
Influenza virus A/Puerto Rico/8/34 (H1N1, PR8)	kindly provided by Dr. Stephan Ludwig, Institute of Molecular Virology, University of Muenster, Germany	N/A
Influenza virus A/Wisconsin/67/2005 (H3N2)	kindly provided by Dr. Stephan Ludwig, Institute of Molecular Virology, University of Muenster, Germany	N/A

**Chemicals, peptides and recombinant proteins**		

Brain-heart-infusion broth	Sigma	Cat#53286
d4-LTB_4_	Cayman Chemical	Cat#Cay320110; CAS: 124629-74-9
d4-PGE_2_	Cayman Chemical	Cat#Cay314010; CAS: 34210-10-1
d5-LXA_4_	Cayman Chemical	Cat#Cay10007737; CAS: 1622429-53-1
d5-RvD2	Cayman Chemical	Cat#Cay11184; CAS: 1881277-33-3
d8-5S-HETE	Cayman Chemical	Cat#Cay334230; CAS: 330796-62-8
d8-AA	Cayman Chemical	Cat#Cay390010; CAS: 69254-37-1
DHA	Cayman Chemical	Cat#90310; CAS: 6217-54-5
Eagle's Minimum Essential Medium	Sigma	Cat#M4526
Fetal calf serum (FCS)	Sigma	Cat#F7524
GM-CSF	Peprotech	Cat#300-23; GenPept: P04141
IFNγ	Peprotech	Cat#300-02; GenPept: P01579.1
IL-4	Peprotech	Cat#200-04; GenPept: P05112
lipopolysaccharide (*E. coli*)	Sigma	Cat#L3129
Histopaque®-1077	Sigma	Cat#10771
M-CSF	Peprotech	Cat#300-25; GenPept: P09603
methyl formate	Sigma	Cat#291056; CAS: 107-31-3
penicillin/streptomycin	Sigma	Cat#P0781
RPMI 1640	Sigma	Cat#R8758

**Experimental models: Cell lines**		

MDCK II cells	kindly provided by Dr. Stephan Ludwig, Institute of Molecular Virology, University of Muenster, Germany	N/A

**Critical commercial assays**		

PerfeCTaTM SYBR® Green SuperMix, ROXTM kit	Quantabio, Beverly	Cat#95055
High-Capacity cDNA Reverse Transcription Kit with RNase Inhibitor	Thermo Fisher Scientific	Cat#4374966
E.Z.N.A® Total RNA Kit 1	Omega Bio-tek	Cat#R6834
Human Anti-Virus response panel (LEGENDplex)	Biolegend	Cat#740349

**Software and algorithms**		

Data Analysis Software Suite for LEGENDplex™	BioLegend	https://www.biolegend.com/en-us/immunoassays/legendplex/support/software
FlowJo X Software	BD Biosciences	https://www.flowjo.com/solutions/flowjo/downloads
Analyst software 1.6.3	AB Sciex	https://sciex.com/products/software/analyst-software
GraphPad Prism 8	GraphPad Software Inc	https://www.graphpad.com/scientific-software/prism/
Odyssey 3.0 software	LI-COR	https://www.licor.com/bio/products/software/image_studio/index.html
OriginPro 2021	OriginLab Corporation	https://www.additive-net.de/de/software/produkte/originlab/originpro

### Resource availability

#### Lead contact

Further information and requests for resources and reagents should be directed to and will be fulfilled by the lead contact, Dr. Oliver Werz (oliver.werz@uni-jena.de).

#### Materials availability

This study did not generate new unique reagents. Antibodies, reagents, cell lines and animals used for experiments were obtained from commercial or internal sources as reported in the key resources table.

#### Data and code availability

• Data: This paper did not generate any omics dataset. All data reported in this paper will be shared by the lead contact upon request.
• Code: This paper does not report original code.
• Any additional information required to reanalyze the data reported in this paper is available from the lead contact upon request.

### Experimental model and study participant details

#### Monocyte isolation and macrophage differentiation and polarization

Monocytes were isolated from leukocyte concentrates obtained from freshly withdrawn peripheral blood of healthy adult human donors which were provided by the Institute of Transfusion Medicine at the University Hospital Jena, Germany. The experimental protocol was approved by the ethical committee of the University Hospital Jena. All methods were performed in accordance with the relevant guidelines and regulations. Peripheral blood mononuclear cells (PBMC) were separated from the leukocyte concentrates using dextran sedimentation of erythrocytes, followed by centrifugation on lymphocyte separation medium (Histopaque®-1077, Sigma-Aldrich). PBMC were seeded in RPMI 1640 (Sigma-Aldrich) containing 10% (v/v) heat-inactivated fetal calf serum (FCS), 100 U/mL penicillin, and 100 μg/mL streptomycin in cell culture flasks (Greiner Bio-one, Frickenhausen, Germany) for 1.5 h at 37°C and 5% CO_2_ for adherence of monocytes. For differentiation of monocytes to macrophages and polarization towards M1- and M2-like phenotypes, published criteria were used.^54^ M1 were generated by incubating monocytes with 20 ng/ml GM-CSF (Peprotech, Hamburg, Germany) for 6 days in RPMI 1640 supplemented with 10% FCS, 2 mmol/L glutamine (Biochrom/Merck, Berlin, Germany), and penicillin-streptomycin (Biochrom/Merck), followed by 100 ng/ml LPS and 20 ng/ml IFN-γ (Peprotech) treatment for another 48 h. For obtaining M2, monocytes were incubated with 20 ng/ml M-CSF (Peprotech) for 6 days and then with 20 ng/ml IL-4 (Peprotech) for additional 48 h.

#### Bacterial strains

The following bacterial strains were used: USA300 wildtype (*S. aureus*/USA300) (a gift from Dr. Lorena Tuchscherr, Institute of Medical Microbiology, Jena University Hospital, Germany) and GFP-expressing *S. aureus* (*S. aureus*/USA300/GFP) (a gift from Dr. Oliwia Makarewicz, Institute of Infectious Diseases and Infection Control, Jena University Hospital, Germany). For experiments with intact *S. aureus*, bacteria were grown overnight at 37°C in brain heart infusion (BHI) medium while shaking, diluted to OD_600nm_ of 0.05 and grown for another 3 h (log-phase). Bacteria were washed in PBS and resuspended in RPMI medium.

#### Viral strains

The influenza virus A/Puerto Rico/8/34 (H1N1, PR8), originally isolated from humans and the human influenza virus A/Wisconsin/67/2005 (H3N2) (a gift from Dr. Stephan Ludwig, Institute of Virology, University of Muenster, Germany) and propagated and passaged in Madin Darby canine kidney (MDCK II) cells (a gift from Dr. Stephan Ludwig, Muenster, Germany). MDCK II cells were cultivated in Eagle's Minimum Essential Medium (EMEM, Sigma-Aldrich, Germany) supplemented with 10% FCS (Sigma Aldrich, USA) at 37°C and 5% CO_2_. To determine the number of infectious particles in the supernatant of the indicated samples, standard plaque assays were performed. For this, MDCK II cells were seeded in 6-well plates until a 90% confluence and subsequently were infected with serial dilutions of the supernatants in infection-PBS (PBS with 1 mM MgCl_2_, 0.9 mM CaCl_2_ and 0.2% BSA) for 30 min at 37°C. After aspiration of the inoculum, cells were incubated with 2 mL medium containing soft agar (MEM with 0.9% agar (Oxoid, Wesel, Germany), 0.01% DEAE-Dextran (Pharmacia Biotech, Germany), 0.2% BSA, 0.2% NaHCO_3_ (Sigma-Aldrich, Germany) and 0.25 μg/ml trypsin-TPCK) at 37°C and 5% CO_2_ for 3 days. Plaque-forming units (PFU) were determined upon cell staining with neutral red (Sigma, Aldrich, Germany).

### Method details

#### Incubations of macrophages and LM metabololipidomics

Polarized M1- and M2-MDM (2 × 10^6^/mL) were incubated in PBS (supplemented with 0.2% BSA, 1 mM MgCl_2_ and 0.9 mM CaCl_2_) with IAV H1N1 (PR8; MOI of 5) for 30 min at 37°C. Then, the cells were washed with PBS and *S. aureus* at MOI of 10 or vehicle were added in RPMI 1640 for another 4 h at 37°C. After the indicated incubation periods, the supernatants were transferred to 2 mL of ice-cold methanol containing 10 μL of deuterium-labeled internal standards (200 nM d8-5S-HETE, d4-LTB_4_, d5-LXA_4_, d5-RvD2, d4-PGE_2_ and 10 μM d8-AA; Cayman Chemical/Biomol GmbH, Hamburg, Germany) to facilitate quantification and sample recovery. Sample preparation was conducted by adapting published criteria.^21^ In brief, samples were kept at −20°C for 60 min to allow protein precipitation. After centrifugation (1200 g, 4°C, 10 min) 8 mL acidified H_2_O was added (final pH = 3.5) and samples were subjected to solid phase extraction. Solid phase cartridges (Sep-Pak® Vac 6cc 500 mg/ 6 mL C18; Waters, Milford, MA) were equilibrated with 6 mL methanol and 2 mL H_2_O before samples were loaded onto columns. After washing with 6 mL H_2_O and additional 6 mL *n*-hexane, LM were eluted with 6 mL methyl formate. Finally, the samples were brought to dryness using an evaporation system (TurboVap LV, Biotage, Uppsala, Sweden) and resuspended in 100 μL methanol-water (50/50, v/v) for UPLC-MS-MS automated injections. LM profiling was analyzed with an Acquity™ UPLC system (Waters, Milford, MA, USA) and a QTRAP 5500 Mass Spectrometer (ABSciex, Darmstadt, Germany) equipped with a Turbo V™ Source and electrospray ionization. LM were eluted using an ACQUITY UPLC® BEH C18 column (1.7 μm, 2.1 × 100 mm; Waters, Eschborn, Germany) at 50°C with a flow rate of 0.3 ml/min and a mobile phase consisting of methanol-water-acetic acid of 42:58:0.01 (v/v/v) that was ramped to 86:14:0.01 (v/v/v) over 12.5 min and then to 98:2:0.01 (v/v/v) for 3 min^20^ The QTrap 5500 was operated in negative ionization mode using scheduled multiple reaction monitoring (MRM) coupled with information-dependent acquisition. The scheduled MRM window was 60 sec, optimized LM parameters were adopted,^55^ and the curtain gas pressure was set to 35 psi. The retention time and at least six diagnostic ions for each LM were confirmed by means of external standards (Cayman Chemical/Biomol GmbH, Hamburg, Germany). Quantification was achieved by calibration curves for each LM. Linear calibration curves were obtained for each LM and gave r^2^ values of 0.998 or higher (for fatty acids 0.95 or higher). Additionally, the limit of detection for each targeted LM was determined.

#### Analysis of cytokine release

Measurement of cytokines was performed by using multiplex bead-based immunoassays (LEGENDplex, BioLegend, San Diego, CA, USA).^56^ Briefly, supernatants from M1- and M2-MDMs after infection with *S. aureus* and/or H1N1 were collected (see: Incubations of macrophages and LM metabololipidomics). 50 μL of supernatant of each sample were measured in duplicate within a 96-well plate by using the BD FACSLyric flow cytometer (BD, Heidelberg, Germany). Data analysis was performed with LEGENDplex™ data analysis software from BioLegend (San Diego, CA, USA).

#### RNA preparation, reverse transcription-PCR, and real-time PCR

Total cellular RNA was extracted using E.Z.N.A® Total RNA Kit 1 (Omega Bio-tek, Norcross, GA, USA), and the isolated RNA was reverse transcribed into cDNA with High-Capacity cDNA Reverse Transcription Kit with RNase Inhibitor (Thermo Fisher Scientific, Waltham, MA, USA) according to the manufacturer’s instructions. The cDNA was mixed with PerfeCTaTM SYBR® Green SuperMix, ROXTM kit (Quantabio, Beverly, MA, USA), and the real-time PCR was performed on a qTOWER3G touch Instrument (Analytic Jena, Jena, Germany). The primers used for the real-time PCR are listed in Table S1.

#### SDS-PAGE and western blot

Cell lysates of MDM corresponding to 2 × 10^6^ cells were separated on 16% polyacrylamide gels for mPGES-1 and 10% polyacrylamide gels for COX-1 and COX-2. Gels were blotted onto nitrocellulose membranes (Amersham™ Protran Supported 0.45 μm nitrocellulose, GE Healthcare, Freiburg, Germany). The membranes were incubated with the following primary antibodies: rabbit polyclonal anti-COX-1, 1:1000 (#4841, Cell Signaling, Danvers, MA); rabbit monoclonal anti-COX-2, 1:1000 (D5H5, #12282, Cell Signaling); rabbit monoclonal anti-mPGES-1, 1:1000 (EPR13765, #ab180589, Abcam, Cambridge, UK) and mouse monoclonal anti-β-actin, 1:1000 (8H10D10, #3700, Cell Signaling). Immunoreactive bands were stained with following secondary antibodies: IRDye 800CW Goat anti-Rabbit IgG (H + L), 1:15 000 (926–32211, LI-COR Biosciences, Lincoln, NE); and IRDye 680LT Goat anti-Mouse IgG (H + L), 1:40 000 (926–68020, LI-COR Biosciences), and visualized by an Odyssey infrared imager (LI-COR Biosciences). Data from densitometric analysis were background corrected.

#### Analysis of bacterial phagocytosis

To determine intracellular bacterial titers *S. aureus*/USA300/GFP were used. After the indicated time points, supernatant was removed and cells were washed with PBS and incubated with PBS plus 5 mM EDTA to detach macrophages for 20 min at 37°C. Cells were centrifuged (400 g, 5 min, RT) and then fixed with 4% paraformaldehyde for 20 min. Intracellular GFP fluorescence was measured by using the BD FACSLyric flow cytometer (BD, Heidelberg, Germany). Data analysis was performed with FLOWJO (BD).

#### Measurement of bacterial colony-forming units (CFUs)

To determine extracellular bacterial titers, supernatants of MDM incubations (100 μL) were plated on brain-heart infusion (BHI) agar plates and incubated overnight at 37°C. CFUs were counted and calculated. Each experiment was performed in technical duplicates.

#### Measurement of extracellular virus

At the indicated time points, supernatants of MDM incubations were collected to assess the number of infectious particles by standard virus plaque assay. Briefly, the Madin-Darby canine kidney (MDCK ΙΙ) cell line was used for this purpose and grown in MEM to 90% confluence in six-well dishes. Cells were washed and infected with serial dilutions of the supernatants in PBS/BA (PBS containing 0.2% BSA, 1 mM MgCl_2_, 0.9 mM CaCl_2_, 100 U/mL penicillin and 0.1 mg/mL streptomycin) for 30 min at 37°C and 5% CO_2_. The inoculum was aspirated and cells were overlaid with 2 mL MEM containing 0.2% BSA, 1 mM MgCl_2_, 0.9 mM CaCl_2_, 100 U/mL penicillin and 0.1 mg/mL streptomycin supplemented with 0.6% agar (Oxoid, Hampshire, United Kingdom), 0.3% DEAE-dextran (Amersham Pharmacia Biotech, Freiburg, Germany), and 1.5% NaHCO_3_ (Gibco Invitrogen, Karlsruhe, Germany). After incubation at 37°C and 5% CO_2_ for 2 to 3 days, virus plaques were visualized by staining with neutral red (Sigma-Aldrich, Munich, Germany).

### Quantification and statistical analysis

Results are expressed as means ± standard error of the mean (SEM) of n observations, where n represents the number of experiments with separate donors, performed on different days, as indicated. Analyses of data were conducted using GraphPad Prism 8 software (San Diego, CA). Two-tailed t test was used for comparison of two groups. For multiple comparison, one-way analysis of variance (ANOVA) with Dunnett's or Tukey’s post hoc tests were applied as indicated. The criterion for statistical significance is *p* < 0.05.
