# Supplementary material for: Influenza A virus selectively elevates prostaglandin E2 formation in pro-resolving macrophages
Source: iScience. 2023 Dec 26;27(1):108775. doi: 10.1016/j.isci.2023.108775 (PMC10797193; doi:10.1016/j.isci.2023.108775)
Supplement: Document S1. Figures S1–S3, Table S1, and Data S1 [file mmc1.pdf]

iScience, Volume *27*

Supplemental information

Influenza A virus selectively elevates prostaglandin E_2 formation in pro-resolving macrophages

Paul M. Jordan, Kerstin Günther, Vivien Nischang, Yuping Ning, Stefanie Deinhardt-Emmer, Christina Ehrhardt, and Oliver Werz

Supplementary Figure S1

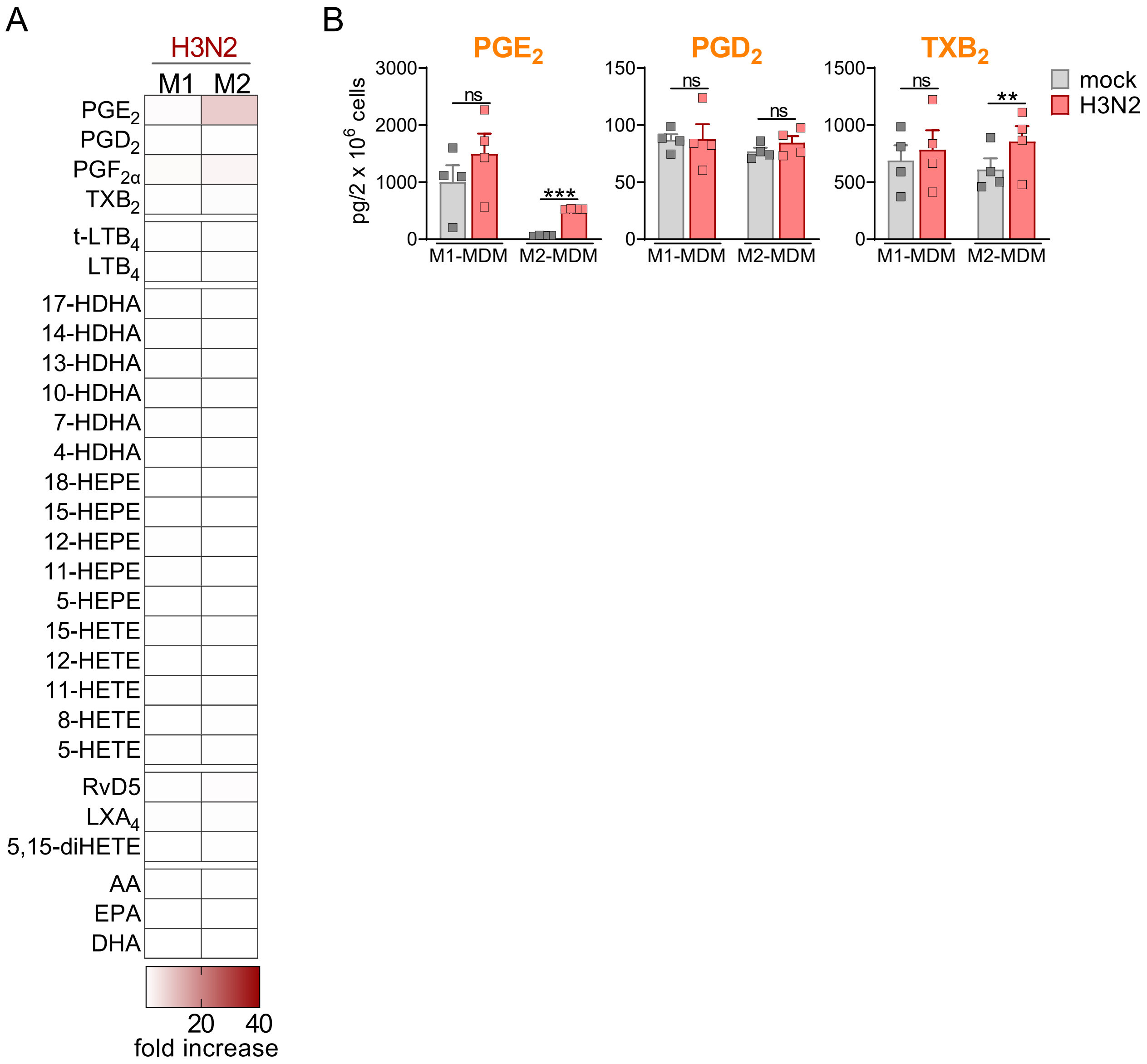

Supplementary Figure S1. H3N2 induces specific PGE_2 formation in M2-MDMs, Related to Figure 2.
Human M1- or M2-MDM (2×10^6 cells) were infected with H3N2 (A/Wisconsin/67/2005 ; MOI = 5) or mock for 30 min, then washed, and further incubated for 4 h at 37 °C. Formed LMs in the supernatants were extracted by SPE and analyzed by UPLC-MS-MS. Data are presented in heatmaps showing the -fold change to mock control (**A**). In (**B**), COX-mediated LMs, given as pg/2×10^6 mock- or H3N2-infected MDMs, are shown as bar charts; data are means + SEM with single values; n = 4. Data were log-transformed for statistical analysis; $^{**}p < 0.01$; $^{***}p < 0.001$; versus mock-infected MDMs; ratio-paired t-test.

Supplementary Figure S2

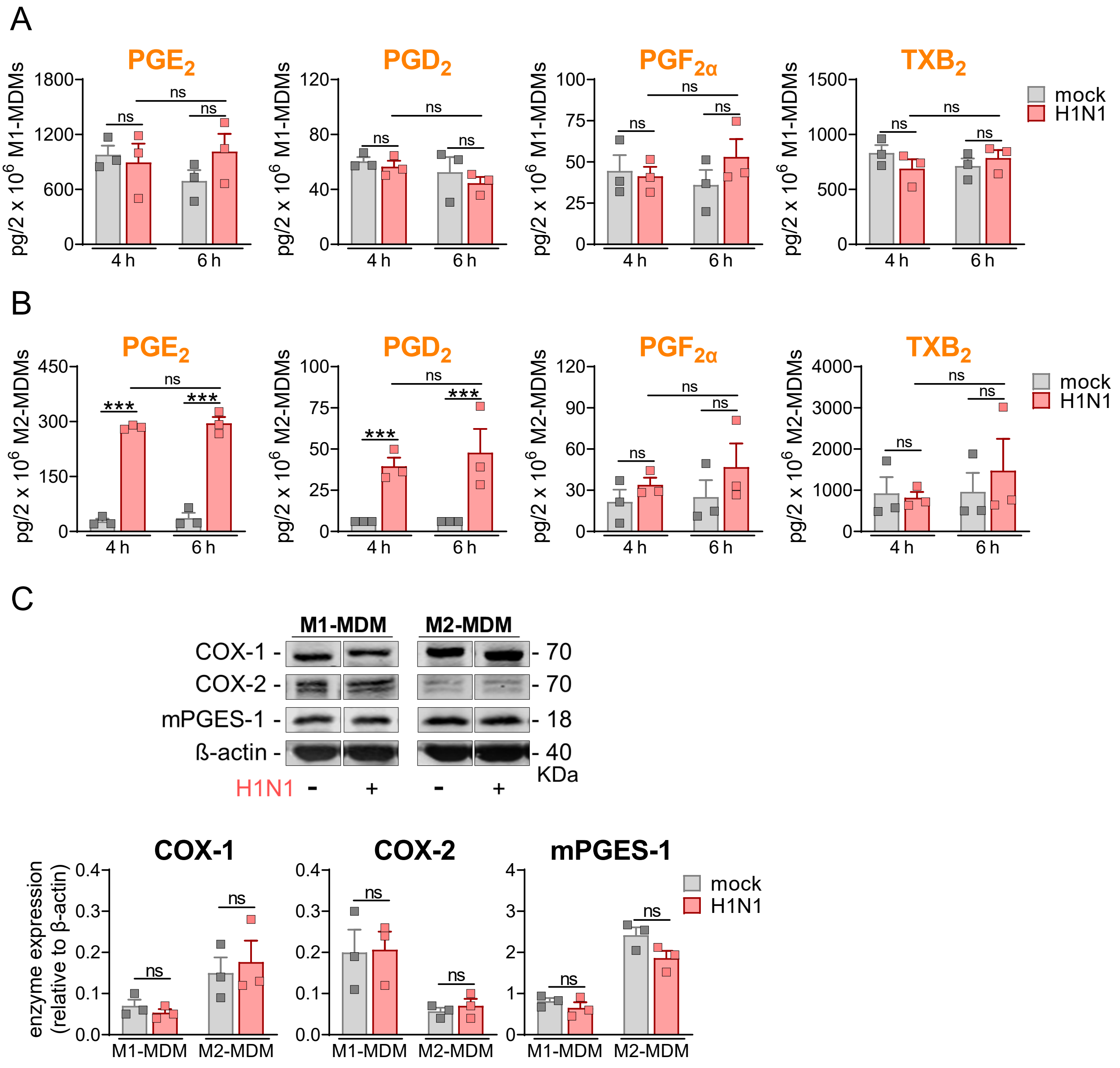

Supplementary Figure S2. The induction of PGE_2 did not increase over time, Related to Figure 2.
(**A-C**) Human M1- or M2-MDMs (2×10^6 cells) were infected with H1N1 (PR8; MOI = 5) or mock for 30 min, then washed, and further incubated for 4 h or 6 h at 37 °C. (**A,B**) Formed LMs in the supernatants were extracted by SPE and analyzed by UPLC-MS-MS. COX products are given as pg/2 × 10^6 cells as single values with mean + SEM for M1-MDMs (**A**) and for M2-MDMs (**B**) ***p < 0.001; Two-way ANOVA with Tukey's multiple comparisons test, $n = 3$. (**C**) Cells were harvested after 6 h and immunoblotted for COX-1, COX-2 and mPGES-1 enzyme expression and normalized to β-actin for densitometric analysis. Exemplary results and densitometric analysis are shown. Data are presented as single values plus means and SEM for M1- and M2-MDMs; ratio-paired t-test. n = 3.

Supplementary Figure S3

fold: 0 – 1 – 10

		pg/ 2x10^6 M1-MDMs mock	H1N1	*S. aureus*	coinfection	coinfection vs. H1N1 fold	*p*	coinfection vs. *S. aureus* fold	*p*
COX	PGE_2	141 ± 58	257 ± 49	933 ± 458	1,249 ± 504	4.9	*	1.3	
	PGD_2	6.1 ± 0.6	9.6 ± 0.3	21 ± 7.3	25 ± 8.6	2.6		1.2	
	$PGF_{2\alpha}$	20 ± 2.3	27 ± 2.9	101 ± 33	135 ± 41	4.9	*	1.3	*
	TXB_2	385 ± 17	375 ± 30	1,830 ± 591	2,257 ± 579	6.0	*	1.2	
5-LOX	LTB_4	17 ± 3.0	12 ± 2.1	462 ± 310	599 ± 423	52		1.3	
	t-LTB_4	12 ± 2.2	10 ± 2.1	83 ± 51	111 ± 78	11	*	1.3	
monohydroxylated PUFAs	17-HDHA	49 ± 6.5	41 ± 2.2	48 ± 9	57 ± 18	1.4		1.2	
	14-HDHA	7.6 ± 2.8	8 ± 3.3	12 ± 6	15 ± 7.4	2.0	*	1.3	
	13-HDHA	5.7 ± 2.0	7.9 ± 2.4	14 ± 7.0	18 ± 9.8	2.3		1.3	***
	10-HDHA	4.7 ± 1.7	4.6 ± 1.5	8.8 ± 3.4	9 ± 3.8	2.0	*	1.0	
	7-HDHA	34 ± 3.4	32 ± 1.9	41 ± 2.6	42 ± 4.3	1.3		1.0	
	4-HDHA	6.4 ± 2.3	4.9 ± 1.5	15 ± 6.7	14 ± 5.8	2.9	*	1.0	
	18-HEPE	7.7 ± 2.4	7.8 ± 2.5	16 ± 8.8	18 ± 8.8	2.3	*	1.1	
	15-HEPE	14 ± 0.4	15 ± 1.3	14 ± 3.8	16 ± 3.3	1.1		1.1	
	12-HEPE	11 ± 1.9	11 ± 0.2	12 ± 2.0	14 ± 3.6	1.3		1.1	
	11-HEPE	2.4 ± 0.9	3.1 ± 1.2	8.4 ± 4.9	10 ± 6.2	3.3	**	1.2	
	5-HEPE	5.3 ± 0.8	5.3 ± 1.9	41 ± 26	51 ± 38	9.6	**	1.3	
	15-HETE	36 ± 8.6	42 ± 18	151 ± 87	174 ± 109	4.1	*	1.2	
	12-HETE	12 ± 3.3	16 ± 5.8	31 ± 17	35 ± 17	2.2	**	1.1	
	11-HETE	13 ± 3.9	17 ± 8.0	114 ± 79	135 ± 95	8.1	**	1.2	*
	8-HETE	17 ± 0.7	16 ± 1.3	31 ± 9.0	30 ± 11	1.9		0.9	
	5-HETE	45 ± 6.8	77 ± 39	309 ± 166	368 ± 217	4.8	**	1.2	
SPMs	RvD5	n.d.	n.d.	n.d.	n.d.				
	LXA_4	4.7 ± 0.3	5.2 ± 0.2	5.5 ± 0.5	6.3 ± 0.4	1.2		1.1	
	5,15-diHETE	7.9 ± 1.9	6.4 ± 2.1	27 ± 11	47 ± 22	7.4		1.8	
PUFAs	AA	5,218 ± 656	4,046 ± 916	26,485 ± 13,445	28,243 ± 18,245	7.0		1.1	
	EPA	910 ± 168	757 ± 247	5,535 ± 3,115	5,834 ± 4,310	7.7		1.1	
	DHA	5,083 ± 1,112	4,121 ± 1,196	14,826 ± 5,184	14,372 ± 6,935	3.5	*	1.0	*

Supplementary Figure S3. Lipid mediator profiles of M1-MDMs after infection and coinfection, Related to Figure 4. Human M1-MDMs (2 × 10^6 cells) were infected with H1N1 (PR8; MOI = 5) or mock for 30 min, washed, and then treated with *S. aureus* (MOI = 10) or mock for 4 h at 37 °C, as indicated. Released LMs of M1-MDMs in the supernatants were assessed by UPLC-MS-MS. Results of mock, single H1N1 and *S. aureus* infection and co-infection with H1N1 and *S. aureus* are shown in pg/2 × 10^6 cells as mean ± SEM, and the fold from H1N1/*S. aureus*-treated samples versus H1N1 or *S. aureus* single infection are given and visualized in a heatmap. Data were log-transformed for statistical analysis; *$p < 0.05$; **$p < 0.01$; ***$p < 0.01$ ratio-paired t-test; coinfected MDMs versus H1N1-infected MDMs or S. aureus-infected MDMs, $n = 3$.

Supplementary Table S1

gene name	Protein name	Sense (5’-3’)	Anti-sense (5’-3’)
COX1	*COX-1*	TCTTGCTGTTCCTGCTCCTG	GTCACACTGGTAGCGGTCAA
COX2	*COX-2*	TGCCTGATGATTGCCCGACT	TGAAAGCTGGCCCTCGCTTA
MPGES1	*mPGES-1*	GGAACGACATGGAGACCATC	GGAAGACCAGGAAGTGCATC
PTGDS	*L-PGDS*	ACCAGTGTGAGACCCGAACC	CAGCGCGTACTGGTCGTAGT
ALOX5	*5-LOX*	ACCCACCTTCTGCGAACACA	GTGGCGTTGGCCTTGTCAAA
ALOX5AP	*FLAP*	CTGCGTTTGCTGGACTGATG	GGAGATGGTGGTGGAGATCG
ALOX15	*15-LOX-1*	CTTCAAGCTTATAATTCCCCAC	GATTCCTTCCACATACCGATAG
ALOX15B	*15-LOX-2*	CTACAGGCTGGCTCTGCTTT	GGATCAGGACAGGGTTGAGA
IL1B	*IL-1β*	TTCGAGGCACAAGGCACAAC	TTCACTGGCGAGCTCAGGTA
IL6	*IL-6*	TTCGGTACATCCTCGACGGC	TCTGCCAGTGCCTCTTTGCT
TNF	*TNF-α*	ACTTTGGAGTGATCGGCCCC	TGGGCTACAGGCTTGTCACT
ACTB	*β-actin*	ACAGAGCCTCGCCTTTGCC	CCATCACGCCCTGGTGCC

Supplementary Table S1. Primer sequences of qPCR analysis, Related to Fig. 2.
Listed are gene name and protein name with specific primer sequences.

Supplementary Data S1

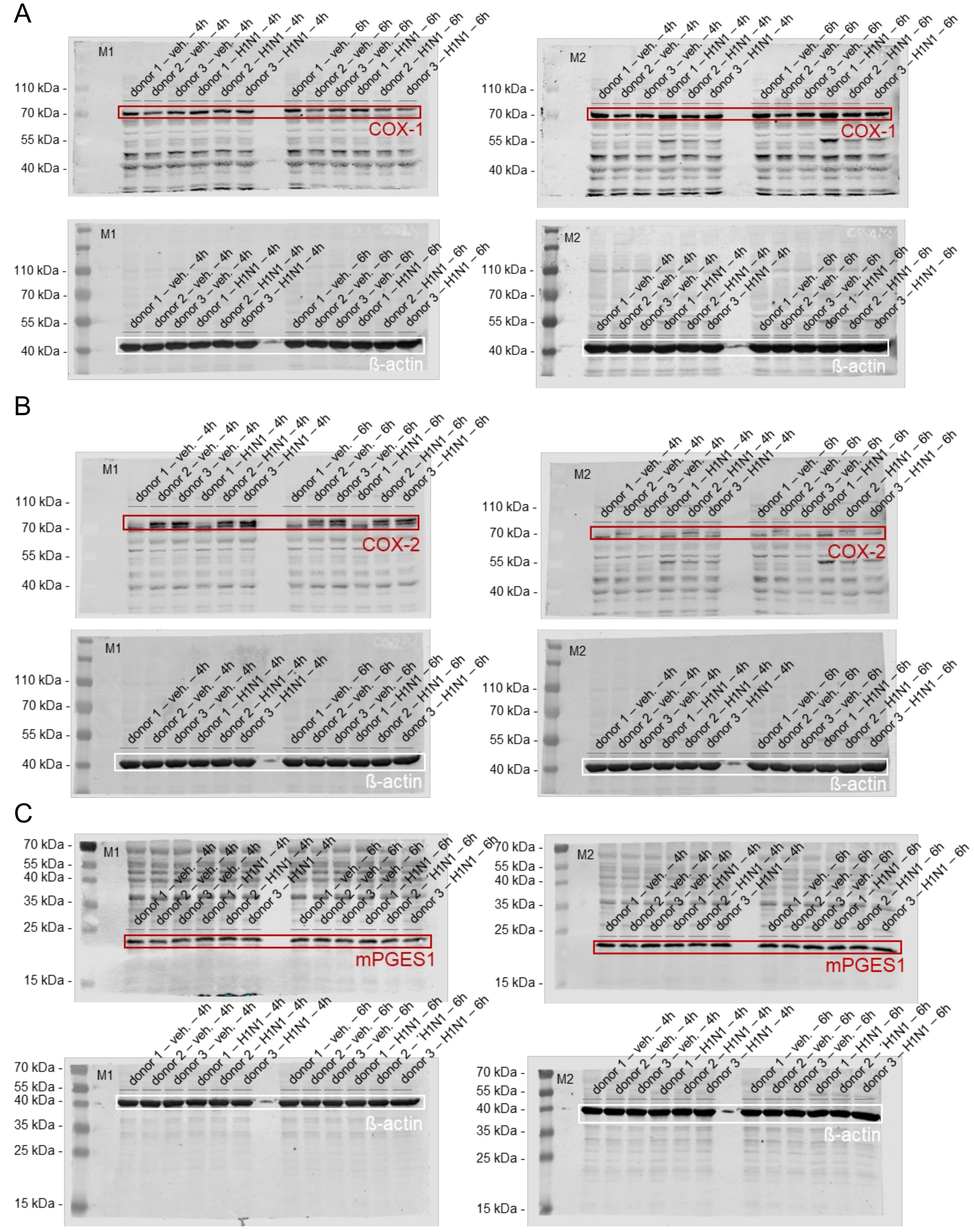

Supplementary Data S1. Raw Western blots, Related to Figure 2 and Supplementary Figure 2. Single, uncropped Western Blots of COX-1 (**A**), COX-2 (**B**) and mPGES-1 (**C**) in M1- and M2-MDMs exposed to H1N1 or mock for 4 or 6 h; n = 3.
